# Effects of Air Pollution and the Introduction of the London Low Emission Zone on the Prevalence of Respiratory and Allergic Symptoms in Schoolchildren in East London: A Sequential Cross-Sectional Study

**DOI:** 10.1371/journal.pone.0109121

**Published:** 2015-08-21

**Authors:** Helen E. Wood, Nadine Marlin, Ian S. Mudway, Stephen A. Bremner, Louise Cross, Isobel Dundas, Andrew Grieve, Jonathan Grigg, Jeenath B. Jamaludin, Frank J. Kelly, Tak Lee, Aziz Sheikh, Robert Walton, Christopher J. Griffiths

**Affiliations:** 1 MRC-PHE Centre for Environment and Health, King’s College London, London, United Kingdom; 2 Asthma UK Centre for Applied Asthma Research, Centre for Primary Care and Public Health, Blizard Institute, Queen Mary University of London, London, United Kingdom; 3 Blizard Institute, Barts and the London School of Medicine and Dentistry, Queen Mary University of London, London, United Kingdom; 4 Institute for Research in Molecular Medicine (INFORMM), Universiti Sains Malaysia, Kelantan, Malaysia; 5 MRC & Asthma UK Centre in Allergic Mechanisms of Asthma, King’s College London, London, United Kingdom; 6 Allergy Centre, HK Sanatorium and Hospital, Hong Kong, China; 7 Allergy & Respiratory Research Group, Centre for Population Health Sciences, The University of Edinburgh, Edinburgh, United Kingdom; 8 Division of General Internal Medicine and Primary Care, Brigham and Women's Hospital/Harvard Medical School, Boston, Massachusetts, United States of America; Nanjing University, CHINA

## Abstract

The adverse effects of traffic-related air pollution on children’s respiratory health have been widely reported, but few studies have evaluated the impact of traffic-control policies designed to reduce urban air pollution. We assessed associations between traffic-related air pollutants and respiratory/allergic symptoms amongst 8–9 year-old schoolchildren living within the London Low Emission Zone (LEZ). Information on respiratory/allergic symptoms was obtained using a parent-completed questionnaire and linked to modelled annual air pollutant concentrations based on the residential address of each child, using a multivariable mixed effects logistic regression analysis. Exposure to traffic-related air pollutants was associated with current rhinitis: NOx (OR 1.01, 95% CI 1.00–1.02), NO_2_ (1.03, 1.00–1.06), PM_10_ (1.16, 1.04–1.28) and PM_2.5_ (1.38, 1.08–1.78), all per μg/m^3^ of pollutant, but not with other respiratory/allergic symptoms. The LEZ did not reduce ambient air pollution levels, or affect the prevalence of respiratory/allergic symptoms over the period studied. These data confirm the previous association between traffic-related air pollutant exposures and symptoms of current rhinitis. Importantly, the London LEZ has not significantly improved air quality within the city, or the respiratory health of the resident population in its first three years of operation. This highlights the need for more robust measures to reduce traffic emissions.

## Introduction

A growing number of studies have shown an association between traffic-related pollution and respiratory and allergic symptoms in children [1–7]. The recent Health Effects Institute review of the evidence relating traffic-related air pollution to health concluded that there is sufficient evidence to link traffic with exacerbation of asthma symptoms in children, and suggestive evidence for a link to asthma onset in childhood [8].

Levels of traffic-related air pollution in London are among the worst in Europe, with European Union (EU) limit values for particulate matter with an aerodynamic diameter of <10μm (PM_10_) and nitrogen dioxide (NO_2_) regularly exceeded in many areas of the city. The London Low Emission Zone (LEZ) was established in February 2008, with the aim of reducing traffic-related air pollution, thereby improving air quality and the health of Londoners. It is the largest such zone in the world, covering an area of 1,600 km^2^ with a population of 8.2 million [9]. The LEZ affects larger diesel vehicles (i.e. vans, trucks, buses, etc, but not cars and taxis), which must meet EU emission standards to enter the zone, or pay a penalty fine. It was introduced in phases between 2008 and 2012, progressively affecting more vehicles and with stricter emissions limits.

A study undertaken to model the expected impact of the LEZ on London’s air quality prior to its implementation found that traffic-related PM_10_ emissions would be reduced by 2% in 2008 and 6% in 2012, and nitrogen oxide (NO_x_) emissions would be reduced by 4% in 2010 and 10% in 2012 [10]. Greater reductions were predicted in areas where concentrations of PM_10_ and/or NO_2_ exceeded EU limit values.

The current study was undertaken to assess the accrued health benefits of living within the LEZ on the respiratory health of schoolchildren in the east London boroughs of Hackney and Tower Hamlets. Within these boroughs, a number of ‘hot spots’ were identified where the LEZ was predicted to have a significant effect on concentrations of PM_10_ and/or NO_2_ [11]. We studied cross-sectional samples of 8–9 year-old children from schools located at varying distances from these ‘hot spots’, during the first three years of the LEZ. We hypothesized that reduced exposure to traffic emissions would result in a reduction in the prevalence of respiratory/allergic symptoms associated with traffic-related pollutants. It was logistically impossible to include a comparable control group in a low air pollution area, and to begin data collection before the LEZ was introduced. However, our study did encompass a period of increasing dieselization of the vehicle fleet [12] and so provides an important update to current knowledge.

## Methods

### Study population and design

Schools in Tower Hamlets and Hackney were selected to achieve a range of annual exposures to NO_2_ (based on dispersion models [10]). These two east London Boroughs were selected due to having ‘hot spots’ of high air pollution levels and predictions that the LEZ would have a significant impact on air quality in these areas [11]. Information on the characteristics of these two Boroughs are included in the S1 Table. Schools were approached and asked to participate; in those which agreed, all Year 4 children (aged 8–9) were invited to take part. This was a sequential cross-sectional study, with data collected over three consecutive winters (Nov-Mar, 2008–11). Health questionnaires were distributed to all Year 4 children. During each study visit, health assessments were conducted to examine lung function and collect biological samples, and completed questionnaires were collected.

### Ethics Statement

Parents were required to give their written consent and children their written and verbal assent, to participate in the health assessment. A flowchart of study participation is shown in the S1 Fig. The study was approved by the local research ethics committee (East London & The City HA Local Research Ethics Committee 2, REC Ref Number 08-H0704-139) and conformed to the standards set by the Declaration of Helsinki.

### Health outcome assessment

Information was collected through a parent-completed questionnaire (S1 Text). Respiratory /allergic symptoms were assessed using questions from the validated International Study of Asthma and Allergies in Childhood (ISAAC) questionnaire for 6–7 year olds [13]. The current and lifetime respiratory/allergic symptoms considered in the analysis are defined in the S2 Table. Information on the questionnaires was entered as recorded, regardless of apparent inconsistencies and whether or not the instructions were followed. Unanswered questions were coded as ‘missing’. Symptom prevalence was calculated by dividing the number of positive responses by the total number of completed questionnaires.

Additional information on sex, age, ethnicity and residential address was obtained from school records. Socioeconomic status was assigned according to residential address using the Index of Multiple Deprivation 2010 [14]. Height and weight were measured during the health assessment and body mass index (BMI) was calculated. Spot urine samples were collected to determine environmental tobacco smoke (ETS) exposure. Urinary cotinine concentration was measured by ELISA (Product number M155B1, Concateno, Abingdon UK) and corrected for creatinine (Product number 500701, Cayman Chemical Company, Ann Arbor, MI, USA). Samples with a cotinine:creatinine ratio (CCR) of ≥30 ng/mg were defined as positive for ETS exposure [15].

### Exposure assessment

Annual concentrations of NOx, NO_2_, PM_10_ and PM with an aerodynamic diameter of <2.5μm (PM_2.5_) were obtained from dispersion models for Greater London, with separate models for each of the three years of data collection. All exposures were based on the annual mean within a 20m buffer zone around the residential address of each subject (further details of the model are presented in the S2 Text). A comprehensive description of this model has been published previously, together with information on validation against measurements [16] and its performance against other urban dispersion models [17]. A description of the contribution of traffic, and of traffic emissions targeted by the LEZ is included in the S3 Text.

### Statistical analyses

Statistical analyses were performed using STATA v.10.1. Models were examined at the 5% significance level for two-sided tests. Details of the power calculations used to derive the necessary sample size are outlined in the S4 Text. Respiratory / allergic symptoms were recorded as binary variables. Associations between air pollution and other factors that could potentially confound the association with health outcomes were assessed by simple logistic regression analysis obtaining the crude odds ratio (OR) with the 95% confidence interval (CI). Adjusted analyses for associations between each air pollutant and respiratory/allergic health outcomes were performed using multilevel mixed effects logistic regression analyses adjusted for age, sex, BMI, IMD score, ethnicity and ETS exposure and allowing a random effect for school. If the observed outcome frequency for confounding variables was too low, only a subset of the covariates was added to the model to avoid over-fitting. Study years were also included as coefficients in the model to account for any potential year-on-year changes.

## Results

A total of 1808 children at the 23 participating schools were invited to take part. 1018 undertook the health assessment, and of these 901 returned a completed questionnaire. In addition, questionnaires were returned by 94 children who declined to undertake the health assessment. Hence, while 56% of children participated in the study, 89% of those who participated returned a questionnaire. The participants are described in Table 1. Information on sex and ethnicity was missing for a small number of children (1.7 and 1.4%, respectively).

**Table 1:** Prevalence of current respiratory/allergic symptoms among all children, and by sex and ethnicity.

	Total	Wheeze	Rhinitis	Eczema
All (%)	995 (100)	111 (11.2)	242 (24.3)	148 (14.9)
Sex				
Male (%)	497 (49.9)	70 (14.1)	139 (30.0)	69 (13.9)
Female (%)	481 (48.3)	40 (8.3)	100 (20.8)	76 (15.8)
Not specified (%)	17 (1.7)	1 (5.9)	3 (17.6)	3 (17.6)
Ethnicity				
Asian (%)	360 (36.2)	34 (9.4)	96 (26.7)	57 (15.8)
Black (%)	240 (24.1)	24 (10.0)	51 (21.3)	45 (18.8)
White (%)	268 (26.9)	41 (15.3)	62 (23.1)	31 (11.6)
Mixed/other (%)	113 (11.4)	12 (10.6)	29 (25.7)	12 (10.6)
Not specified (%)	14 (1.4)	0	4 (28.6)	3 (21.4)

Percentages are for rows, except for first column which reads vertically (e.g. 49.9% of all respondents were male; 14.1% of males have current wheeze); percentages may not add to 100.0 due to rounding

The prevalence of current wheeze, rhinitis and eczema symptoms is shown for all children in Table 1. 11.2% of children reported current symptoms of wheeze. Severe wheeze symptoms were reported by 5.5% of children, with 3.9% reporting ≥4 attacks of wheezing in the last year; however, among children with current wheeze, the prevalence of severe symptoms was high: 29.7% had ≥4 attacks in the last year, 24.3% had ≥1 night/week of disturbed sleep, and 21.6% had wheeze-limited speech, suggesting poor control of symptoms or lack of asthma diagnosis. Dry cough at night was reported by 26.2%. Current wheeze was negatively associated with age (OR 0.41, 95% CI 0.21–0.82, p = 0.011), positively associated with BMI (1.07, 1.00–1.14, p = 0.041), was lower in children of Asian background compared with White children (0.46, 0.26–0.81, p = 0.008), and was less common in girls than boys (0.47, 0.30–0.74, p = 0.001) (see Table 2), as was severe wheeze (0.32, 0.12–0.84, p = 0.021).

**Table 2:** Odds ratios for associations of potentially confounding variables with prevalence of current respiratory/allergic symptoms.

	Wheeze	Rhinitis	Eczema
	(n = 111)	(n = 242)	(n = 148)
Age	0.42 (0.21 to 0.82)*	1.11 (0.68 to 1.80)	1.01 (0.55 to 1.84)
Sex (Female vs. Male)	0.47 (0.30 to 0.74)**	0.69 (0.50 to 0.94)*	1.14 (0.77 to 1.68)
BMI (kg/m^2^)	1.07 (1.00 to 1.14)*	1.06 (1.01 to 1.11)*	1.01 (0.95 to 1.07)
ETS exposure	1.14 (0.66 to 1.95)	1.01 (0.67 to 1.52)	0.95 (0.56 to 1.61)
IMD score	1.01 (0.99 to 1.04)	1.00 (0.99 to 1.02)	0.99 (0.98 to 1.01)
Ethnicity (Asian vs. White)	0.46 (0.26 to 0.81)**	1.19 (0.78 to 1.80)	1.69 (0.98 to 2.93)
Ethnicity (Black vs. White)	0.55 (0.30 to 1.01)	0.88 (0.56 to 1.40)	1.85 (1.05 to 3.29)*
Ethnicity (mixed/other vs. White)	0.73 (0.35 to 1.50)	1.22 (0.71 to 2.10)	0.90 (0.41 to 1.98)
Study year (Yr 2 vs. Yr 1)	0.97 (0.52 to 1.80)	0.97 (0.61 to 1.56)	0.90 (0.49 to 1.65)
Study year (Yr 3 vs. Yr 1)	0.60 (0.31 to 1.16)	0.69 (0.43 to 1.13)	1.02 (0.56 to 1.87)

Data shown as odds ratio (OR) for unit increase in variable unless otherwise stated, with 95% confidence intervals in brackets; ORs adjusted for all variables shown in table; ETS exposure = positive urinary cotinine:creatinine ratio (CCR ≥ 30ng/mg);

* p<0.05

**p<0.01

Current symptoms of rhinitis were reported by 24.3% of children, with 21% reporting that their daily activities were affected; but among children with current rhinitis, 75% were affected to some extent. Current rhinitis was less common in girls than boys (OR 0.69, 95% CI 0.50–0.94, p = 0.020) and was positively associated with BMI (1.06, 1.01–1.11, p = 0.012).

Current eczema symptoms were reported by 14.9% of children, with 4.6% reporting ≥1 night/week of disturbed sleep, increasing to 18.9% in children with current eczema. In 37.2% of those with current eczema, their itchy rash had not cleared during the last year. Eczema symptoms had most commonly first occurred since the age of 5 (41.2% of those with current eczema), although for 35.8% they had first occurred when the child was <2 years old. Current eczema was higher in Black than White children (OR 1.85, 95% CI 1.05–3.29, p = 0.035).

Lifetime asthma, hay fever and eczema were reported by 14.4%, 27.6, and 25.4% of children, respectively. Lifetime asthma was reported by only 66.7% of children who reported current symptoms of wheeze. Lifetime asthma was negatively associated with age (OR 0.49, 95% CI 0.27–0.90, p = 0.021), positively associated with BMI (1.07, 1.01–1.13, p = 0.021) and with IMD score (1.02, 1.00–1.04, p = 0.042) and was less common in girls than boys (0.62, 0.42–0.93, p = 0.019) (see Table 3). Lifetime asthma was lower in Year 3 compared with Year 1 of the study (OR 0.51, CI 0.29–0.90, p = 0.020), decreasing significantly from 21.2% in Year 1 to 14.5% in Year 2 to 12.2% in Year 3 (Fishers exact test, p = 0.038).

**Table 3:** Odds ratios for associations of potentially confounding variables with prevalence of lifetime (ever having had) asthma, hay fever and eczema.

	Asthma	Hay fever	Eczema
	(n = 143)	(n = 275)	(n = 253)
Age	0.49 (0.27 to 0.90)*	0.99 (0.62 to 1.59)	1.41 (0.85 to 2.35)
Sex (Female vs. Male)	0.62 (0.42 to 0.93)*	1.03(0.76 to 1.40)	1.19 (0.86 to 1.64)
BMI (kg/m^2^)	1.07 (1.01 to 1.13)*	1.00 (0.95 to 1.04)	1.03 (0.98 to 1.09)
ETS exposure	1.40 (0.87 to 2.27)	1.11 (0.74 to 1.65)	0.57 (0.36 to 0.90)*
IMD score	1.02 (1.00 to 1.04)*	0.99 (0.98 to 1.01)	0.99 (0.98 to 1.01)
Ethnicity (Asian vs. White)	0.67 (0.39 to 1.16)	2.08 (1.36 to 3.19)**	0.45 (0.28 to 0.72)**
Ethnicity (Black vs. White)	0.92 (0.52 to 1.63)	2.09 (1.32 to 3.30)**	0.76 (0.48 to 1.21)
Ethnicity (mixed/other vs. White)	1.37 (0.72 to 2.57)	2.09 (1.22 to 3.59)**	0.55 (0.31 to 0.96)*
Study year (Yr 2 vs. Yr 1)	0.64 (0.37 to 1.10)	1.11 (0.68 to 1.81)	1.43 (0.83 to 2.48)
Study year (Yr 3 vs. Yr 1)	0.51 (0.29 to 0.90)*	1.25 (0.76 to 2.04)	1.22 (0.70 to 2.13)

Data shown as odds ratio (OR) for unit increase in variable unless otherwise stated, with 95% confidence intervals in brackets; ORs adjusted for all variables shown in table; ETS exposure = positive urinary cotinine:creatinine ratio (CCR ≥ 30ng/mg);

* p<0.05

**p<0.01

Among those children with current rhinitis, 48% reported having had hay fever. Hay fever was twice as high in children of all other ethnicities compared with White children (Asian OR 2.08, 95% CI 1.36–3.19, p = 0.001; Black 2.09, 1.32–3.30, p = 0.002; mixed/other 2.09, 1.22–3.59, p = 0.008).

Among those children with current symptoms of eczema, 62.2% reported having ever had eczema. The prevalence of lifetime eczema was lower in children of Asian (OR 0.45, 95% CI 0.28–0.72, p = 0.001) and of mixed/other (0.55, 0.31–0.96, p = 0.037) ethnic backgrounds compared with White children, and was negatively associated with ETS exposure (0.57, 0.36–0.90, p = 0.015).

Air pollutant concentrations changed little during the study (Table 4), based on data obtained from sites within the London Air Quality Network [18] in and surrounding the study area. For PM_2.5_ all sites across London were included, due to the more limited monitoring. For both NOx and NO_2_ there was no evidence of a reduction at either urban background or roadside locations. Similarly, PM_2.5_ and PM_10_ concentrations were unaltered at background sites over the first three years of the LEZ. Some evidence of a roadside decrease in PM_10_ was noted between 2008 and 2009, but only when measurements based on Tapered Element Oscillating Microbalance (TEOM) masses were employed (p = 0.027), with a similar trend in PM_2.5_ TEOM concentrations (p = 0.06). Similar reductions were not apparent between 2009 and 2010. These small changes were not reflected in the modelled exposure attributions based on the children’s residential addresses (Table 5).

**Table 4:** Measured annual mean pollutant concentrations (μg/m^3^) at selected background (Bk) and roadside (RS) sites surrounding the study area.

	Site type	NOx	NO_2_	PM_10_	PM_2.5_ (FDMS)	PM_2.5_ TEOM)
**Year 1 2008**	Bk	68.4±19.9 (40.2–101.5, n = 8)	41.1±8.7 (26.0–52.6, n = 8)	22.0±1.5 (20.7–24.9, n = 8)	No data	10.5±0.4 (10.3–11.0, n = 3)
	RS	135.9±41.1 (89.8–223.0, n = 13)	57.2±10.8 (43.7–70.3, n = 13)	28.7±6.1 (20.3–40.9, n = 11)	17.1±1.2 (16.0–18.5, n = 4)	14.9±3.3 (11.5–20.2, n = 8)
**Year 2 2009**	Bk	66.1 ± 22.1 (36.9–105.2, n = 8)	40.8 ± 10.1 (23.5–57.2, n = 8)	22.5 ± 1.91 (20.4–25.8, n = 6)	14.7 ± 1.8 (13.2–17.6, n = 6)	10.3 ± 0.9 (9.7–11.6, n = 4)
	RS	131.9 ± 47.4 (87.2–255.7, n = 14)	56.8 ± 12.1 (44.2–82.3, n = 14)	26.9 ± 5.1* (20.5–36.9, n = 11)	15.1 ± 3.1 (11.7–19.0, n = 4)	14.2 ± 3.0 (11.1–18.6, n = 7)
**Year 3 2010**	Bk	67.4 ± 23.4 (36.8–100.6, n = 6)	40.5 ± 10.5 (24.3–48.6, n = 6)	21.9 ± 0.5 (21.5–22.7, n = 5)	14.7 ± 1.4 (12.8–16.5, n = 7)	10.4 ± 1.4 (9.4–12.5, n = 4)
	RS	131.7 ± 40.2 (82.7–220.3, n = 14)	56.7 ± 11.7 (41.8–74.0, n = 14)	26.2 ± 3.6 (19.9–32.6, n = 12)	17.6 ± 1.7 (15.1–19.9, n = 5)	13.9 ± 2.1 (11.4–16.4, n = 4)

Annual mean ± SD pollutant concentrations (range, number of sites included), based on sites within and surrounding the London boroughs of Hackney and Tower Hamlets. Annual means were calculated for each of the included sites with greater than 75% data capture across the given year. PM_10_ is expressed as reference equivalent concentration, based on the correction of Tapered Element Oscillating Microbalance (TEOM) masses for the loss of volatile components using the volatile correction method [30] and combined with measurements made using the Filter Dynamics Measurement System (FDMS). As no empirical method is available for correcting PM_2.5_ TEOM masses, the TEOM and FDMS mass concentrations are given separately. The average annual mean concentrations across each of the site types was compared using a one way ANOVA, with post hoc testing performed using paired t-tests: *, significant reduction (p<0.05) in pollutant concentration between 2008 and 2009

**Table 5:** Annual mean levels of air pollutants at residential address, by year and averaged across study period, μg/m^3^.

	n	NOx	NO_2_	PM_10_	PM_2.5_
Year 1 (2008)	131*	71.0 ± 10.6 (61.5–122.8)	41.9 ± 3.9 (38.4–61.0)	22.9 ± 1.3 (21.7–29.8)	13.0 ± 0.5 (12.5–16.0)
Year 2 (2009)	418*	79.3 ± 13.1 (50.8–235.4)	44.9 ± 4.9 (32.9–98.9)	23.9 ± 1.2 (20.7–32.7)	14.4 ± 0.5 (12.6–18.0)
Year 3 (2010)	444	73.8 ± 16.4 (47.1–220.8)	42.7 ± 6.1 (30.9–90.8)	23.0 ± 1.5 (20.4–33.2)	13.3 ± 0.6 (12.0–17.3)
Years 1–3	993	75.7 ± 14.8 (47.1–235.4)	43.5 ± 5.5 (30.9–98.9)	23.4 ± 1.5 (20.4–33.2)	13.7 ± 0.8 (12.0–18.0)

Values are mean ± SD (range);

* air pollution data were not available for two participants (one in each of Years 1 and 2 of the study) because their residential addresses were outside the Greater London area

**Fig 1:**
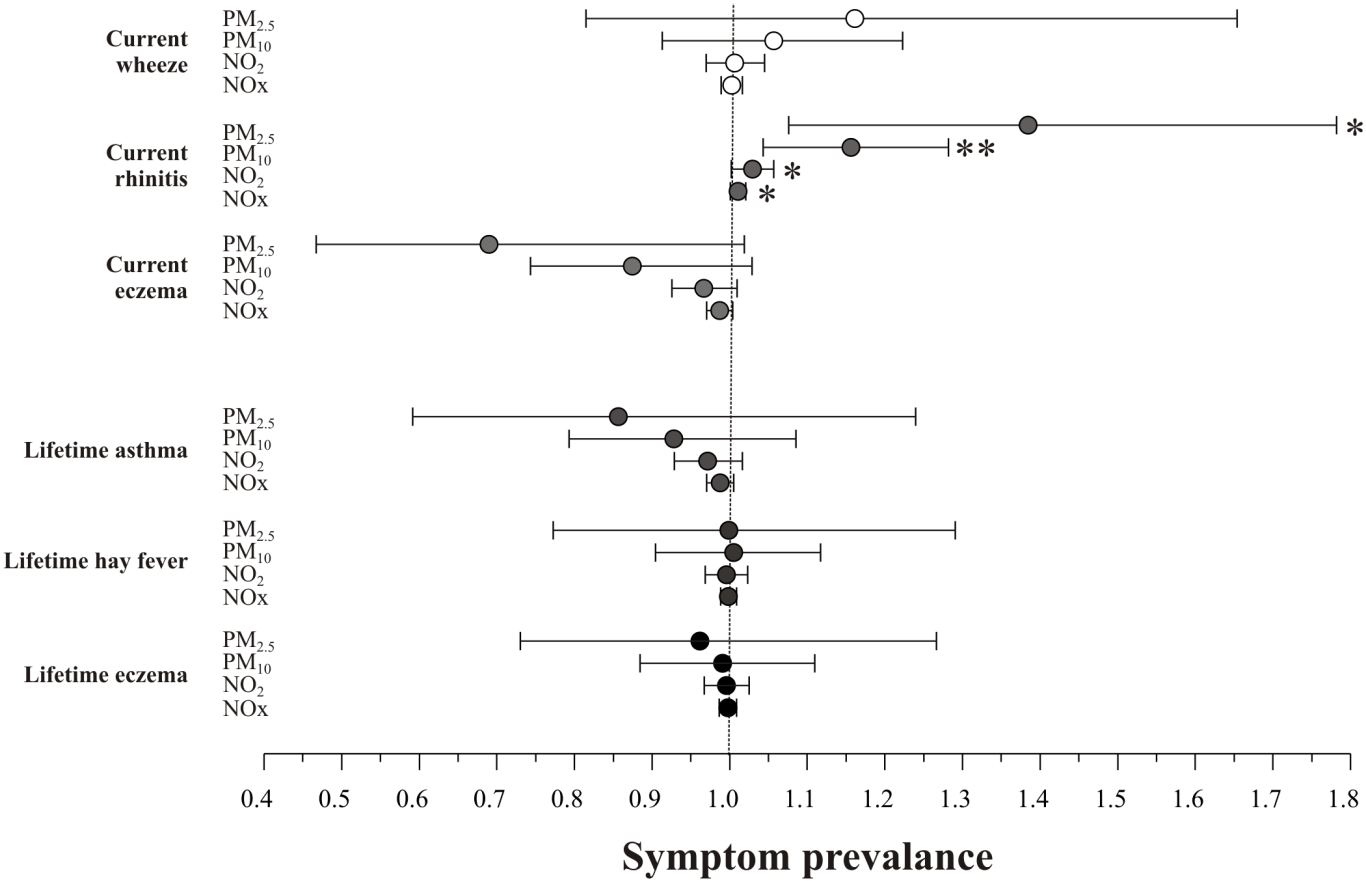
Exposure to air pollution as a risk factor for current and lifetime respiratory/allergic symptoms. Adjusted associations between air pollutants and the prevalence of current and lifetime respiratory/allergic symptoms. Odds ratios adjusted for age, sex, BMI, socio-economic deprivation (IMD score), ETS exposure and year of study, with a random effect for school. Single-pollutant models were calculated for each air pollutant. Odds ratios are for unit increase in pollutant, in μg/m^3^. Current symptoms defined as within the last 12 months; lifetime conditions defined as ‘having ever had’ asthma, hay fever or eczema. Vertical dotted line indicates null (OR = 1). Horizontal lines indicate 95% confidence intervals of odds ratios. * p<0.05, **p<0.01

The prevalence of current rhinitis was positively associated with annual mean NOx (OR 1.01, 95% CI 1.00–1.02, p = 0.033), NO_2_ (1.03, 1.00–1.06, p = 0.034), PM_10_ (1.16, 1.04–1.28, p = 0.006) and PM_2.5_ (1.38, 1.08–1.78, p = 0.011) (Fig 1 and S3 Table, ORs for an increase of 1 μg/m^3^). The prevalence of current wheeze and eczema symptoms and of lifetime asthma, hay fever and eczema was not associated with air pollution levels.

## Discussion

The present study addressed the allergic and respiratory symptoms of children residing within London’s LEZ, over the first three years of its operation. Of the symptoms examined, only current rhinitis was positively associated with annual modelled NOx, NO_2_, PM_10_ and PM_2.5_ exposures and we observed no evidence of a reduction in symptom prevalence over the study period. However, we also failed to observe the improvements in air quality that were predicted to result from the implementation of the LEZ. We are aware of only one other study which has examined the health benefits of a LEZ [19]. In this study, the effects of two LEZs in Rome were investigated; however, no health parameters were measured. Instead, ‘years of life gained’ were estimated using concentration-response functions derived from cohort studies in the USA and Europe [19]. Residents living along busy roads were predicted to have gained 3.4 days/person as a result of the zone being implemented.

Our results are consistent with data from Phase I of the ISAAC study, which concluded that modelled urban background PM_10_ has little or no association with the prevalence of childhood asthma, rhinoconjunctivitis or eczema, either within or between countries [20]. However, findings from Phase III of the ISAAC study indicated a positive relationship between symptoms of asthma and allergic disease with traffic-related pollution [1]. In this phase, a question on frequency of truck traffic on the street of residence was included, and a highly significant exposure-response relationship between truck traffic frequency and respiratory/allergic symptoms was found, with the strongest relationship for symptoms of severe asthma [1]. Other studies using self- or parent-reported traffic frequency data from questionnaires have shown positive associations with respiratory/allergic symptoms. In Bochum, Germany, frequency of heavy truck traffic on the street of residence was positively correlated with the prevalence of wheezing and allergic rhinitis among schoolchildren [3], while in Singapore, Zuraimi et al. [2] found significant dose-response relationships between traffic density and asthma and rhinitis symptoms in pre-school children.

A number of studies have used objective measures of traffic density as a proxy for air pollution exposure. In Munich, Germany, an increase of 25,000 cars daily passing through the school district on the main road was associated with a significant increase in the prevalence of recurrent wheezing with dyspnoea and of recurrent dyspnoea in 9–11 year-old schoolchildren [4], while in Haarlem, The Netherlands, children living on busy streets (based on modelled NO_2_ concentrations) reported a higher prevalence of wheeze compared with children living on quiet streets [6]. In Windsor, Canada, roadway density was found to be significantly associated with wheeze (OR 1.23), wheeze with dyspnoea (OR 1.27) and asthma (OR 1.08) in elementary schoolchildren [5]. However, the measure of traffic exposure was only resolved at the level of the child’s neighbourhood, as road density was calculated from the summed length of all roadways within a 200m radius of the home postcode.

Other studies have used data from ambient air pollution monitoring sites to assign exposure at the individual level. Using a validated three-year dispersion model, exposures were assigned to school addresses for 9–11 year-old schoolchildren in the French Six Cities Study [7]. PM_10_ levels were associated with asthma, eczema and allergic rhinitis, and NOx was associated with asthma. A number of studies from The Netherlands have shown significant associations between proxy or actual measures of traffic pollution and respiratory symptoms, but only among subgroups of children with chronic respiratory symptoms [21], or bronchial hyper-responsiveness and allergic sensitization [22, 23].

Differences in study design may explain some of the inconsistencies in reported associations between air pollution and respiratory/allergic symptoms, e.g. differences in study area, measured pollutants or proxy measure of pollutant exposure, wording of symptom questions or study population [24]. A recent meta-analysis has attempted to address these issues by combining the original data from 11 studies on PM_10_ and respiratory symptoms (>45,000 children from 12 countries), and found that symptoms of wheeze and asthma diagnosis were not associated with PM_10_, although there was a weak association with hay fever diagnosis [24]. A previous meta-analysis by the same group focussed on NO_2_ (data from 5 studies, including 24,000 children from 5 countries) and found no overall evidence of associations between ambient NO_2_ and asthma diagnosis or wheeze [25].

In the current study, NO_2_ and PM_10_ levels were within a similar range to most of the studies cited above, at least for urban areas [7, 21, 22, 24, 25]. Interestingly, for 84% of the children in our study, the annual mean residential NO_2_ level was above the London air quality objective (and World Health Organization, WHO, guideline) of 40 μg/m^3^. The objective for PM_10_ (also 40 μg/m^3^) was not exceeded for any of the children. However, air quality guidelines published by WHO in 2005 [26] recommended an annual mean of 20 μg/m^3^ for PM_10_, which was exceeded for all the children in the current study.

Our study had a number of strengths: it targeted areas with high levels of air pollution, where the greatest air quality improvements were predicted to result from the introduction of the LEZ; it was a population survey based on school classes, including children from a wide diversity of ethnic groups; we employed a validated, widely-used questionnaire to measure respiratory/allergic symptoms, and we used a high-resolution air pollution model, providing exposure measurements at the level of residential address.

A limitation of the type of parent-reported symptom data used herein is the possibility of recall bias or exaggeration of symptoms, especially given that parents were told we were investigating the effects of the LEZ on children’s health. However, the symptom prevalences reported here were lower than those from the ISAAC UK study [27, 28]; hence exaggeration does not appear to have been a problem. Our study was also limited by studying children within the narrow age range of 8–9 years. Our aim was to study young children whose lungs are still developing and are therefore most likely to be adversely affected by any damaging effects of exposure to air pollution. We also needed our subjects to be sufficiently mature to follow instructions and be able to undertake the tests involved in the health assessments, in particular spirometry in order to accurately measure lung function. We believed that 8–9 year-olds best met this requirement. Since our study design was cross sectional, we elected to study children within the narrow age range of a single school year group so that age would not be a significant factor accounting for differences in lung function and other outcome variables. While not necessarily representative of all children living within the LEZ (which covers a large and extremely diverse area), we believe these children are representative of those living in areas with the highest levels of traffic-related air pollution, and therefore most likely to benefit from improvements in air quality. Since we only studied children, our findings are relevant only to children and cannot be extrapolated to other age groups.

There are several reasons why the predicted air quality improvements from the LEZ have not occurred, including the delay in implementing phase III (originally scheduled for October 2010, which would have applied the Euro III PM standard to light goods vehicles), the increasing proportion of diesel cars within the fleet [12], and evidence that NOx emissions from newer diesel engines (Euro 3–5) have not fallen as predicted by the current emission inventories [29]. As the delayed phase III was introduced with phase IV (requiring heavy goods vehicles, buses and coaches to meet the Euro IV PM standard) in January 2012, it is feasible that the predicted improvements may only become apparent in the subsequent years, although this is critically dependent on the emission technologies delivering under real-world driving conditions. Our study is on-going for a further three years, permitting continued analysis of any LEZ-related effects on air quality and subsequent changes in the prevalence of respiratory/allergic symptoms.

In conclusion, this study shows that traffic-related air pollutants have adverse effects on respiratory/allergic symptoms in schoolchildren in London and that London’s LEZ has had no beneficial effect on these symptoms, up to three years after its implementation. As the majority of children in this study are exposed to levels of air pollutants higher than those recommended by the WHO [26], this is an important finding. It is of significant relevance to policy makers to note that the LEZ, which was designed to reduce traffic-related emissions in London, has not actually done so up to this point. In part, for PM_10_ and PM_2.5_ this may reflect the fact that traffic sources (excluding cars and non-exhaust sources, not targeted by the LEZ) only contribute 3.0 and 2.3%, respectively, of the measured concentrations at urban background sites (S3 Text). Hence, any change in PM is likely to have been small and very difficult to detect amongst all of the other 'noise' caused by the weather, economy etc. In contrast, had the LEZ performed as predicted one would have expected a measureable decreases in NOx, as a significant proportion of both the roadside (51.8%) and background concentrations (26.5%) was due to vehicle types targeted by the scheme. This was not seen, largely due to the failure of Euro III and IV diesel engines to produce the predicted NOx emissions under real-world driving conditions [29]. As the subsequent phases of the LEZ target a larger proportion of the vehicle fleet in London, with stricter emissions limits, it will be important to investigate whether improvements do occur in the subsequent years.

## Supporting Information

S1 Fig Study flowchart: from recruitment of schools to number of returned completed questionnaires. (PDF)

S1 Table Key characteristics of the London Boroughs of Hackney and Tower Hamlets, with comparators for London as a whole, and England. (PDF)

S2 Table Definitions of current and lifetime respiratory/allergic symptoms. (PDF)

S3 Table Odds ratios and 95% confidence intervals for effects of modelled air pollutants on respiratory/allergic symptoms. (PDF)

S1 Text Questionnaire Text. (PDF)

S2 Text Air pollution modelling. (PDF)

S3 Text Contributions of traffic to London urban background and roadside pollutant concentrations. (PDF)

S4 Text Sample size. (PDF)
