# Supplementary material for: Effects of Air Pollution and the Introduction of the London Low Emission Zone on the Prevalence of Respiratory and Allergic Symptoms in Schoolchildren in East London: A Sequential Cross-Sectional Study
Source: PLoS One. 2015 Aug 21;10(8):e0109121. doi: 10.1371/journal.pone.0109121 (PMC4546643; doi:10.1371/journal.pone.0109121)

S1 Fig. Study flowchart: from recruitment of schools to number of returned completed questionnaires.

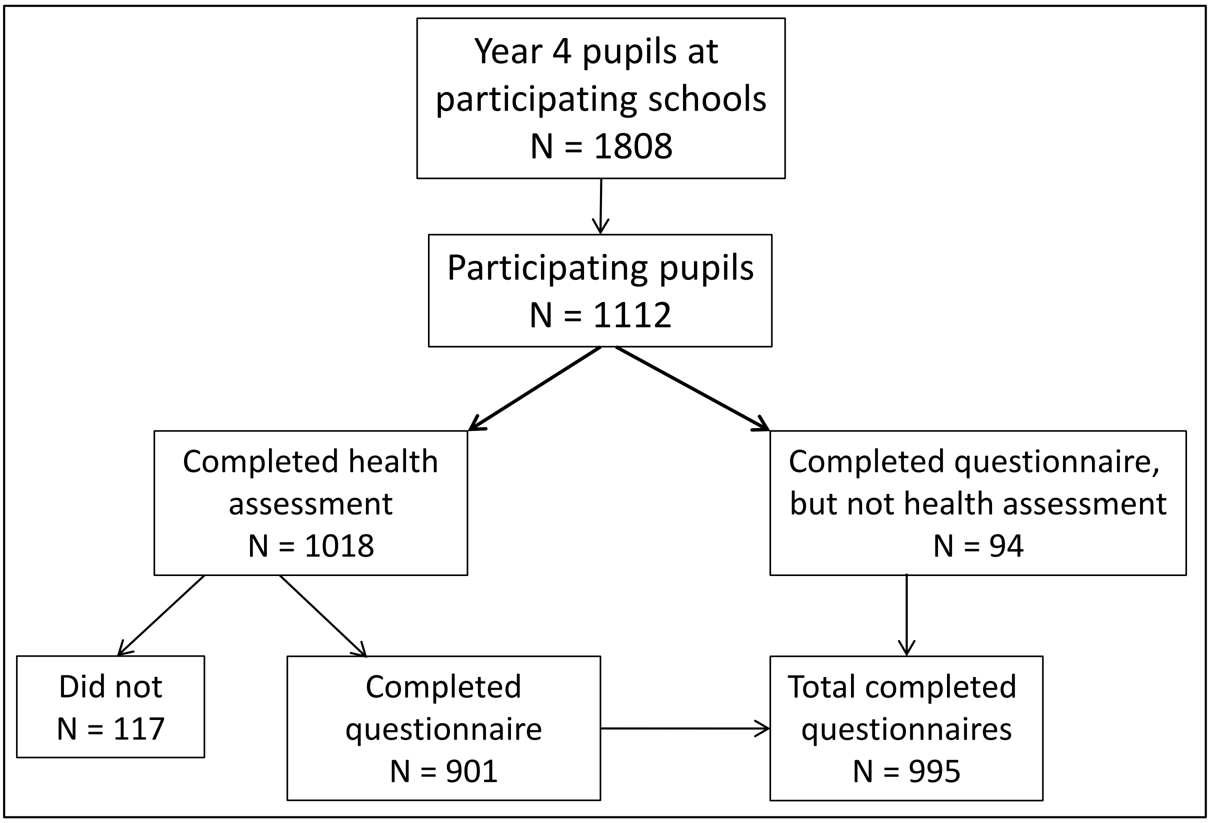

Supplement: S1 Fig — (PDF) [file pone.0109121.s001.pdf]
