# Supplementary material for: Effects of Air Pollution and the Introduction of the London Low Emission Zone on the Prevalence of Respiratory and Allergic Symptoms in Schoolchildren in East London: A Sequential Cross-Sectional Study
Source: PLoS One. 2015 Aug 21;10(8):e0109121. doi: 10.1371/journal.pone.0109121 (PMC4546643; doi:10.1371/journal.pone.0109121)
Supplement: S1 Table — (PDF) [file pone.0109121.s002.pdf]

S1 Table: Key characteristics of the London Boroughs of Hackney and Tower Hamlets, with comparators for London as a whole, and England

Characteristic	Hackney	Tower Hamlets	London	England
Population estimate, 2013	256,600	271,100	8,440,100	13,027,867
Inland Area (Hectares)	1,905	1,978	157,215	53,012,500
Population density (per hectare) 2013	134.7	137.1	53.7	4.1
Proportion of population aged 0-15, 2013	20.5	19.6	20.0	18.9
% of resident population born abroad (2012) [1]	37.2	45.1	35.9	12.4
% of pupils whose first language is not English (2013) [2]	51.0	73.0	43.0	15.6
Largest migrant population by country of birth (2012) [3]	Turkey	Bangladesh	India	India
Indices of Multiple Deprivation 2010 Rank of Average Score[4]	2.0	7.0	-	-

[1] Does not include short term migrants (spending less than a year in the UK); [2] Includes primary and secondary pupils in maintained schools; [3] National comparator is for UK, rather than England; [4] Ranked out of 326 LAs in England (1=most deprived); source for all data in this table: http://data.london.gov.uk/datastore/package/london-borough-profiles, accessed 30/07/2014.
