# Supplementary material for: Effects of Air Pollution and the Introduction of the London Low Emission Zone on the Prevalence of Respiratory and Allergic Symptoms in Schoolchildren in East London: A Sequential Cross-Sectional Study
Source: PLoS One. 2015 Aug 21;10(8):e0109121. doi: 10.1371/journal.pone.0109121 (PMC4546643; doi:10.1371/journal.pone.0109121)
Supplement: S2 Table — (PDF) [file pone.0109121.s003.pdf]

S2 Table: Definitions of current and lifetime respiratory/allergic symptoms

Symptom	Definition
Current	
Wheeze	Has your child had wheezing or whistling in the chest in the past 12 months?
Severe wheeze	Has your child had wheezing or whistling in the chest in the last 12 months? AND AT LEAST ONE OF: ≥4 attacks of wheezing in the past 12 months; ≥1night/week of sleep disturbed by wheezing in the past 12 months; wheezing severe enough to limit speech to only one or two words at a time between breaths in the past 12 months.
Rhinitis	In the past 12 months, has your child had a problem with sneezing, or a runny, or a blocked nose when he/she DID NOT have a cold or the flu?
Eczema	Has your child ever had an itchy rash which was coming and going for at least 6 months? AND Has your child had this itchy rash at any time in the last 12 months? AND Has this itchy rash at any time affected any of the following places: the folds of the elbows, behind the knees, in front of the ankles, under the buttocks, or around the neck, ears or eyes?
Lifetime	
Asthma	Has your child ever had asthma?
Hayfever	Has your child ever had hay fever?
Eczema	Has your child ever had eczema?
