# Supplementary material for: Effects of Air Pollution and the Introduction of the London Low Emission Zone on the Prevalence of Respiratory and Allergic Symptoms in Schoolchildren in East London: A Sequential Cross-Sectional Study
Source: PLoS One. 2015 Aug 21;10(8):e0109121. doi: 10.1371/journal.pone.0109121 (PMC4546643; doi:10.1371/journal.pone.0109121)
Supplement: S3 Table — (PDF) [file pone.0109121.s004.pdf]

S3 Table. Odds ratios and 95% confidence intervals for effects of modelled air pollutants on respiratory/allergic symptoms

**Pollutant**	**Symptom**	**OR (95% CI)**
PM_{10}	Current wheeze	1.06 (0.91-1.22)
	Current rhinitis	1.16** (1.04-1.28)
	Current eczema	0.87 (0.74-1.03)
	Lifetime asthma	0.93 (0.79-1.09)
	Lifetime hay fever	1.01 (0.90-1.12)
	Lifetime eczema	0.99 (0.88-1.11)
$PM_{2.5}$	Current wheeze	1.16 (0.82-1.65)
	Current rhinitis	1.38* (1.08-1.78)
	Current eczema	0.69 (0.47-1.02)
	Lifetime asthma	0.86 (0.59-1.24)
	Lifetime hay fever	1.00 (0.77-1.29)
	Lifetime eczema	0.96 (0.73-1.27)
NO_2	Current wheeze	1.01 (0.97-1.04)
	Current rhinitis	1.03* (1.00-1.06)
	Current eczema	0.97 (0.93-1.01)
	Lifetime asthma	0.97 (0.93-1.02)
	Lifetime hay fever	1.00 (0.97-1.02)
	Lifetime eczema	1.00 (0.97-1.03)
NOx	Current wheeze	1.00 (0.99-1.02)
	Current rhinitis	1.01* (1.00-1.02)
	Current eczema	0.99 (0.97-1.00)
	Lifetime asthma	0.99 (0.97-1.01)
	Lifetime hay fever	1.00 (0.99-1.01)
	Lifetime eczema	1.00 (0.99-1.01)

Odds ratios adjusted for age, sex, BMI, socio-economic deprivation (IMD score), ETS exposure and year of study, with a random effect for school. Single-pollutant models were calculated for each air pollutant. Odds ratios are for unit increase in pollutant, in $\mu g/m^3$, * $p<0.05$, ** $p<0.01$
