# Supplementary material for: Effects of Air Pollution and the Introduction of the London Low Emission Zone on the Prevalence of Respiratory and Allergic Symptoms in Schoolchildren in East London: A Sequential Cross-Sectional Study
Source: PLoS One. 2015 Aug 21;10(8):e0109121. doi: 10.1371/journal.pone.0109121 (PMC4546643; doi:10.1371/journal.pone.0109121)
Supplement: S1 Text — (PDF) [file pone.0109121.s005.pdf]

S1. Questionnaire Text

Core questions for asthma (from ISAAC questionnaire for 6-7 year-olds)

1. Has your child ever had wheezing or whistling in the chest at any time in the past? Yes/No
   If you have answered "No" please skip to question 6
2. Has your child had wheezing or whistling in the chest in the past 12 months? Yes/No
   If you have answered "No" please skip to question 6
3. How many attacks of wheezing has your child had in the past 12 months? None/1-3/4-12/More than 12
4. In the past 12 months, how often, on average, has your child's sleep been disturbed due to wheezing? Never woken with wheezing/Less than one night per week/One or more nights per week
5. In the past 12 months, has wheezing ever been severe enough to limit your child's speech to only one or two words at a time between breaths? Yes/No
6. Has your child ever had asthma? Yes/No
7. In the past 12 months, has your child's chest sounded wheezy during or after exercise? Yes/No
8. In the past 12 months, has your child had a dry cough at night, apart from a cough associated with a cold or chest infection? Yes/No

Core questions for rhinitis (from ISAAC questionnaire for 6-7 year-olds)

1. Has your child ever had had a problem with sneezing, or a runny nose, or blocked nose when he/she did not have a cold or the flu? Yes/No
   If you have answered "No" please skip to question 6
2. In the past 12 months, has your child had a problem with sneezing, or a runny nose, or blocked nose when he/she did not have a cold or the flu? Yes/No
   If you have answered "No" please skip to question 6
3. In the past 12 months, has this nose problem been accompanied by itchy-watery eyes? Yes/No
4. In which of the past 12 months did this nose problem occur? (Please tick any which apply)
   January/February/March/April/May/June/July/August/September/October/November/December
5. In the past 12 months, how much did this nose problem interfere with your child's daily activities? Not at all/A little/A moderate amount/A lot

6. Has your child ever had hay fever? Yes/No

Core questions for eczema (from ISAAC questionnaire for 6-7 year-olds)

1. Has your child ever had had an itchy rash which was coming and going for at least six months? Yes/No
   If you have answered "No" please skip to question 7
2. Has your child had this itchy rash at any time in the past 12 months? Yes/No
   If you have answered "No" please skip to question 7
3. Has this itchy rash at any time affected any of the following places: the folds of the elbows, behind the knees, in front of the ankles, under the buttocks, or around the neck, ears or eyes? Yes/No
4. At what age did this itchy rash first occur? Under 2 years/Age 2-4 years/Age 5 or more
5. Has this rash cleared completely at any time during the past 12 months? Yes/No
6. In the past 12 months, how often, on average, has your child been kept awake at night by this itchy rash? Never in the past 12 months/Less than one night per week/One or more nights per week

Has your child ever had eczema? Yes/No
