# Supplementary material for: Effects of Air Pollution and the Introduction of the London Low Emission Zone on the Prevalence of Respiratory and Allergic Symptoms in Schoolchildren in East London: A Sequential Cross-Sectional Study
Source: PLoS One. 2015 Aug 21;10(8):e0109121. doi: 10.1371/journal.pone.0109121 (PMC4546643; doi:10.1371/journal.pone.0109121)
Supplement: S2 Text — (PDF) [file pone.0109121.s006.pdf]

S2 Text. Air pollution modelling

Annual concentrations of NOx, NO_2, PM_{10} and PM with an aerodynamic diameter of less than 2.5µm ($PM_{2.5}$) were derived using the KCLurban [1] based on the ADMS dispersion model v4 and road source model v2.3 (CERC19), hourly measured meteorological data, empirically derived NO-NO_2-O_3 and PM relationships and emissions from the London Atmospheric Emissions Inventory [2]. Separate annual surface maps for Greater London were produced for 2008, 2009 and 2010 at a 20m x 20m grid point resolution, and linked to the corresponding sampling windows: Nov 2008-Mar 2009, Nov 2009-Apr 2010 and Nov 2010-Apr 2011, respectively. The model for each year took into account a range of pollution sources and emissions, including major and minor road networks, modelled with detailed information on vehicle stock, traffic flows, and speed on a link-by-link basis, as well as pollution sources including large and small regulated industrial processes, boiler plants, domestic and commercial combustion sources, agriculture, rail, ships, airports, and pollution carried into the area by prevailing winds. A comprehensive description of this model has been published previously, together with information on validation against measurements [1] and its performance against other urban dispersion models [3]. All exposures were based on the annual mean within a 20m buffer zone around the residential address of each subject.

References

1. Beevers SD, Kitwiroon N, Williams ML, Kelly FJ, Ross Anderson H, et al. (2013) Air pollution dispersion models for human exposure predictions in London. J Expo Sci Environ Epidemiol.
2. Greater London Authority (GLA) (2008) The London Atmospheric Emissions Inventory 2008.
3. Carslaw DC (2011) Defra Urban Model Evaluation Analysis – Phase 1.
