# Supplementary material for: Effects of Air Pollution and the Introduction of the London Low Emission Zone on the Prevalence of Respiratory and Allergic Symptoms in Schoolchildren in East London: A Sequential Cross-Sectional Study
Source: PLoS One. 2015 Aug 21;10(8):e0109121. doi: 10.1371/journal.pone.0109121 (PMC4546643; doi:10.1371/journal.pone.0109121)
Supplement: S3 Text — (PDF) [file pone.0109121.s007.pdf]

S3 Text. Contributions of traffic to London urban background and roadside pollutant concentrations.

Contribution of traffic to roadside and urban background pollution concentrations in London has been addressed in the recent urban modelling inter-comparison exercise [1]. Focusing on the KCLurban model used for the dispersion modelling in the current paper, traffic contributions to the pollutant concentrations (% by vehicle class) across 16 sites, 8 background and 8 roadside (including sites within the study area) were as follows:

NOx background: Cars (21.6%), LGVs (7.1%), HGVs (8.8%), buses (10.6%). Total traffic contribution: 48.6%. Contribution targeted by the first two phases of the LEZ: 26.5%.
NOx roadside: Cars (28.3%), LGVs (11.1%), HGVs (10.1%), buses (30.6%). Total traffic contribution: 80.1%. Contribution targeted by the first two phases of the LEZ: 51.8%.

PM_{10} background: Cars (1.6%), LGVs (0.5%), HGVs (0.9%), buses (0.4%), vehicle non-exhaust (11.7%). Total traffic contribution: 15.1%. Contribution targeted by the first two phases of the LEZ: 3.0%.
PM_{10} roadside: Cars (3.4%), LGVs (1.2%), HGVs (1.9%), buses (2.1%), vehicle non-exhaust (29.1%). Total traffic contribution: 37.7%. Contribution targeted by the first two phases of the LEZ: 5.2%.

$PM_{2.5}$ background: Cars (2.9%), LGVs (1.1%), HGVs (0.9%), buses (0.3%), vehicle non-exhaust (5.8%). Total traffic contribution: 11.0%. Contribution targeted by the first two phases of the LEZ: 2.3%.
$PM_{2.5}$ roadside: Cars (7.0%), LGVs (2.9%), HGVs (2.3%), buses (2.2%), vehicle non-exhaust (14.7%). Total traffic contribution: 29.1%. Contribution targeted by the first two phases of the LEZ: 7.4%.

Total traffic contribution for PM_{10} and $PM_{2.5}$ includes contributions from non-tailpipe sources, i.e. tire and brake wear. The proportion of the roadside and background contribution targeted by phase I and II of the LEZ are also illustrated, representing the contribution from HGVs, LGVs and buses, but excluding cars and non-exhaust sources. It is not meaningful to talk about the source apportionment of NO_2 because of the chemistry involved, i.e. there is a non-linear relationship between the source emission and concentration. Therefore only NOx figures are quoted though vehicles make a disproportionate contribution due to primary NO_2 – which is higher than other source categories such as gas combustion.

Hence, whilst each phase of the LEZ was predicted to have had a beneficial impact on PM_{10} and NOx, any change in PM is likely to have been small and very difficult to detect amongst all of the other 'noise' caused by the weather, economy etc. This does not preclude effects on primary components of PM. In contrast, had the LEZ performed as predicted one would have expected a measureable decrease in NOx, as a significant proportion of both the roadside and background concentrations was due to vehicle types targeted by the scheme. This was not seen, largely due to the failure of Euro III and IV diesel engines to produce the predicted reductions in NOx emissions under real-world driving conditions [1].

References

1. Carslaw DC (2011) Defra Urban Model Evaluation Analysis – Phase 1.
