# Supplementary material for: Effects of Air Pollution and the Introduction of the London Low Emission Zone on the Prevalence of Respiratory and Allergic Symptoms in Schoolchildren in East London: A Sequential Cross-Sectional Study
Source: PLoS One. 2015 Aug 21;10(8):e0109121. doi: 10.1371/journal.pone.0109121 (PMC4546643; doi:10.1371/journal.pone.0109121)
Supplement: S4 Text — (PDF) [file pone.0109121.s008.pdf]

S4 Text. Sample size

The primary outcome of the study was to detect a 4% improvement year-on-year in the average forced expiratory volume in 1 sec (FEV_1), hence the sample size was determined according to this variable rather than respiratory/allergic symptom prevalence. It was initially determined that 200 children would be needed per year, but this was revised upwards following the first year of data collection. From Year 1 data, the mean FEV_1 was 1.71 litres (SD=0.276 litres) and valid measurements of FEV_1 were obtained from 150/202 i.e. 74% of children. In order to detect a 4% increase in mean FEV_1, from 1.71 to 1.79 litres in two successive years, with 80% power at the 5% significance level, would require 245 children under simple random sampling. Assuming 74% (from Year 1) of children give valid FEV_1 measurements, the cluster size would be 22 children (out of a class of 30). The design effect, based on an intra-cluster correlation coefficient (ICC) of 0.02, is 1 + (22-1)*0.03 = 1.63, inflating the sample size to 399 children per year. Therefore, the number of classes to be sampled in Year 2 and subsequently is 399/22 = 19 classes (rounded up from 18.1).
